# Effects of Straw Return in Deep Soils with Urea Addition on the Soil Organic Carbon Fractions in a Semi-Arid Temperate Cornfield

**DOI:** 10.1371/journal.pone.0153214

**Published:** 2016-04-28

**Authors:** Hongtao Zou, Xuhong Ye, Jiaqi Li, Jia Lu, Qingfeng Fan, Na Yu, Yuling Zhang, Xiuli Dang, Yulong Zhang

**Affiliations:** College of Land and Environment, Shenyang Agricultural University & Key Laboratory of Cultivated Land Conservation, Ministry of Agriculture, Shenyang, PR China; Chinese Academy of Sciences, CHINA

## Abstract

Returning straw to deep soil layers by using a deep-ditching-ridge-ploughing method is an innovative management practice that improves soil quality by increasing the soil organic carbon (SOC) content. However, the optimum quantity of straw return has not been determined. To solve this practical production problem, the following treatments with different amounts of corn straw were investigated: no straw return, CK; 400 kg ha^-1^ straw, S_400_; 800 kg ha^-1^ straw, S_800_; 1200 kg ha^-1^ straw, S_1200_; and 1600 kg ha^-1^ straw, S_1600_. After straw was returned to the soil for two years, the microbial biomass C (MBC), easily oxidized organic C (EOC), dissolved organic C (DOC) and light fraction organic C (LFOC) content were measured at three soil depths (0–10, 10–20, and 20–40 cm). The results showed that the combined application of 800 kg ha^-1^ straw significantly increased the EOC, MBC, and LFOC contents and was a suitable agricultural practice for this region. Moreover, our results demonstrated that returning straw to deep soil layers was effective for increasing the SOC content.

## Introduction

Straw is a relatively abundant resource in China, with an annual straw production of 0.79 billion tons[1]. However, nearly 50% of the straw produced in China is wasted by burning, which results in serious environmental pollution[2]. As one of the most widely protective measures[3–5], returning straw to the soil is beneficial for optimizing the soil environment, preventing and controlling soil degradation, and reducing air pollution that results from burning straw[6–9]. Currently, straw is returned to the soil in the following three ways: mechanical crushing with backward pressure return, direct mulching and leaving tall stubble[10–11]. In China, land must be planted each year, and yearly or quarterly crops are grown overshort intervals to meet food production needs because the amount of available arable land per capita is low. Straw decays slowly because it has a relatively high carbon-to-nitrogen (C/N) ratio, which is not beneficial for the next crop and can decrease the rate of crop emergence[12]. In addition, because straw is in direct contact with the soil, the organic acids in straw increase crop pests and diseases that occur during straw decomposition[13].

In view of the traditional problems with returning straw to the soil, our laboratory developed a method for returning straw to deep soil layers that uses ridge plowing at a depth of 40cm after harvest[14]. When using this new method of straw return to the field, the bottom of the soil plow layer is broken, the soil bulk density is reduced, and the soil structure is improved[15]. Meanwhile, as straw is buried in the furrow, the next crop is planted on the ridge. Thus, the crop roots do not directly contact the straw, which could reduce the incidence of disease caused by the harmful substances produced during straw degradation. However, the optimal amount of straw return using this method has not been determined yet. The amount of straw return is related to multiple factors. Excessive amounts of straw return prevent the complete degradation of straw over a short period, and the addition of insufficient straw does not meet the needs of the carbon pool[16]. Therefore, straw return in actual production systems is an important problem that needs to be addressed.

Soil organic carbon (SOC) content is the most important parameter for sustainable soil use, and the labile fractions of SOC affect soil physical, chemical and biological properties[17–18]. Labile organic carbon (C) significantly impacts soil nutrient transformations, soil formation, microbial metabolism, organic matter decomposition and the migration of soil contaminants[19]. Soil labile organic C fractions, such as microbial biomass C (MBC), easily oxidized organic C (EOC), dissolved organic C (DOC) and light fraction organic C (LFOC), are more sensitive indicators of changes in soil C than the soil total organic carbon (TOC)[20]. Active SOC changes correspond to small changes and occur before total soil carbon changes[21–22]. In addition, SOC turnover is a more sensitive indicator of soil quality and soil fertility and is affected by various tillage systemsand comprehensive soil activity. Furthermore, SOC is necessary for maintaining balance between the soil carbon pool and soil fertility. Thus, we can use the SOC content to determine soil productivity and predict soil environmental changes caused by changes in oil management practices[23].

Straw return is a widely recognized strategy for increasing SOC contents and improving soil quality and crop productivity[24] and increases soil microbial activity and biomass[25]. Dynamic changes in SOC in agricultural soils are mainly determined from the balance between organic material inputs and the degradation rates of existing SOC[26]. Thus, in this paper, we aim to quantify the effects of different amounts of straw return on soil MBC, EOC, DOC, and LFOC contents and determine the optimal amount of straw return at deep soil depths in a semi-arid temperate cornfield.

## Materials and Methods

### Study site

The field experiment was performed at Fumeng County, Fuxin City, Liaoning Province, China (42°10'N, 122°00'E). The owner of the land, Mr. Wang Yi, gave us permission to conduct this study on his land. The site has a semi-arid and semi-humid continental monsoon climate with an average annual rainfall of 450 mm, an average annual evaporation of 1660 mm, a mean annual temperature of 7.6°C,and mean annual sunshine of 2760 h. The frost-free period is 154 days. The soil at the experimental site is silty sand and is classified as cinnamon soil according to the Chinese soil classification system (1992). The main properties of the soil sampled in October 2012 were as follows: bulk density, 1.45 g cm^-3^; organic C, 23.3 g kg^-1^; total nitrogen (N), 1.2 g kg^-1^; nitrate N, 7.4 mg kg^-1^;ammonium N, 10.2 mg kg^-1^; and pH, 7.7.

### Experimental design

After corn harvest in October 2010, the soil was furrowed to a depth of 40cm using deep ditching before covering the soil and ridge plowing to bury the maize straw. The average maize straw production was 9900-1150kg ha^-1^. We designed the straw return experiment to include the following treatments: one-half hectare of straw was returned to one hectare of arable land; one hectare of straw was returned to one hectare of arable land; one and one-half hectare of straw was returned to one hectare of arable land; and two hectares of straw were returned to one hectare of arable land. Straw was buried at a depth of 40 cm at the following concentrations: 400 kg ha^-1^ straw (S_400_), 800 kg ha^-1^ straw (S_800_), 1200 kg ha^-1^ straw (S_1200_), and 1600 kg ha^-1^ straw (S_1600_). The C/N ratio of the straw was 77:1. To achieve a C/N ratio of 25:1[27], additional urea was applied at rates of 1.03 kg ha^-1^, 2.16 kg ha^-1^, 3.09 kg ha^-1^and 4.12 kg ha^-1^, respectively, with a base application of 235 kgha^-1^ urea, 300 kgha^-1^diammonium phosphate and225 kgha^-1^ potassium sulfate before ridge plowing. Thus, the depth of the upper soil containing straw was approximately 20cm. The soil was left undisturbed from the autumn of 2010 to the autumn of 2012. A control treatment (CK) without straw and with the same amount of fertilizer was also studied. The experimental plots were arranged using a random design with three replications, and each plot covered an area of 60 m^2^. The following spring, corn (Zhengdan 958) was cultivated by creating a large double ridge in the ridge-sown maize treatment. Two years later, the straw added at rates of 400 kg ha^-1^ (S_400_) and 800 kg ha^-1^(S_800_) was decomposed completely, and the straw in the other two treatments was partially decomposed. The depth of the upper soil layer above the straw and the depth of the straw were both approximately 10cm. Soil samples were collected and analyzed in October 2012.

### Soil sampling and analysis

Soil samples were collected using “S”-type methods at five points and at three soil depths.(0–10, 10–20 and 20–40 cm). The fresh soil samples were isolated from the straw residues and root debris by passing them through 10-mesh (2 mm) sieves before measuring the organic carbon fractions.

Soil samples were air-dried and passed through a 2 mm sieve for laboratory analysis of soil texture and SOC was measured by Walkley–Black wet oxidation method [28]. The MBC was determined using the chloroform fumigation extraction method[29]. Briefly, a field-moist soil (equivalent to 20 g dry weight) sample with a field moisture capacity of 40%was fumigated with chloroform overnight. After removing the chloroform from the soil sample, C was promptly extracted from the samples using 0.5 mol L^−1^ K_2_SO_4_ at a soil-to-extract ratio of 1:5. The C contents in the K_2_SO_4_-extracted solutions from the chloroform-treated and untreated soils were measured using an automated TOC Analyzer (Shimadzu, TOC-Vcph, Japan). Soil MBC was calculated by subtracting the K_2_SO_4_-extracted C of the untreated soils from theK_2_SO_4_-extracted C of the chloroform-treated soil and calibrated using an extraction efficiency factor (Kc) of 0.38.

The EOC was analyzed via oxidation using 333-mol L^-1^ KMnO_4_[30]. Finely ground air-dried soil samples were oxidized using 25 ml of 333 mM KMnO_4_. The suspensions were horizontally shaken at 60 r min^− 1^ for 1 h and centrifuged at 2000 r min^− 1^ for 5 min. The supernatants were diluted and measured using a spectrophotometer (UV2300) at 565 nm.

DOC was extracted in water at 25°C[20] by shaking the sample and water mixture for 0.5 h at 250 rpm and centrifuging for 10 min at 15,000 rpm. The supernatant liquid was passed through 0.45 μm filter membranes, and the resulting filtrate was stored at −18°C until analysis. The organic C content in the extracts was measured using an automated TOC Analyzer.

LFOC was determined using heavy liquid flotation separation[31]. Three 10 g subsamples of each soil (<2 mm) were weighed in a 100 mL plastic centrifuge tube, and 50 mL of a NaI liquid solution was added to the tube. The tubes were shaken on a shaker for 30 min before centrifuging at 1,000 rpm for 10 min. The floating, light fraction was immediately removed and poured into a plastic bottle. This process was repeated three times, and the supernatant materials from the replicates were poured into the same plastic bottle. The light fraction was collected by passing the solutions through a membrane filter (0.45 μm) in a Büchner funnel and washing three times with0.01 mol L^−1^ CaCl_2_ to remove excess NaI. The material was washed three more times with deionized water before drying at 60°C for 24 h, weighing, and finely grinding it for organic carbon analysis.

### Data analysis

The data were analyzed usingSPSS17.0 (IBM, New York, USA). A single-factor analysis of variance (ANOVA) was used to conduct different comparisons among the five treatments at P<0.05, and the means were separated using least significant difference (LSD).

## Results

### Effects of returning different amounts of straw to deep soil on SOC

**Fig 1:**
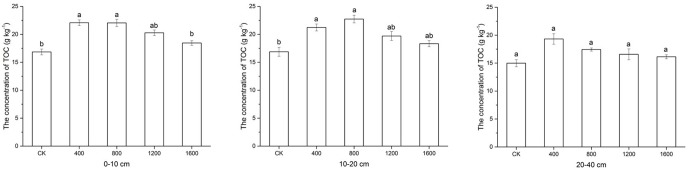
Effects of straw return to the deep soil on soil microbial biomass carbon (SOC) at three soil depths. SOC values are expressed as the means±SE of three replicates. Different letters indicate significant differences among the treatments (*P<0.05).

The changes of the SOC contents in the different soil layers are presented in Fig 1. The same treatment had a small change in three soil layers, with ranges of 19.33–22.07g kg^-1^, 17.43–22.05g kg^-1^, 16.57–20.28g kg^-1^, and 16.15–18.45 g kg^-1^ for S_400_, S_800_, S_1200_, and S_1600_, respectively. S800 had the highest TOC content at a depth of10-20 cm, and the other samples had the highest TOC contents at a depth of 0–10 cm. According to the significant difference test, the TOC contents in S_400_ and S_800_ were significantly higher (P < 0.05) at depths of 10–20 cm and0-10 cm than the CK. No significant difference was observed at a depth of 20–40 cm.

### Effects of returning different amounts of straw to deep soil on MBC

**Fig 2:**
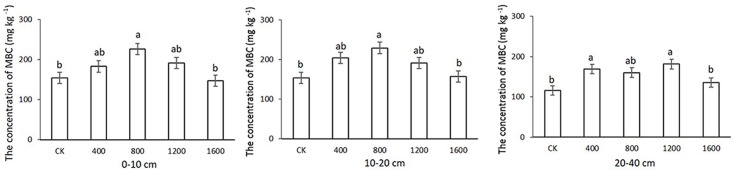
Effects of straw return to the deep soil on soil microbial biomass carbon (MBC) at three soil depths. Values are expressed as the means±SE from three replicates. Different letters indicate significant differences among the treatments(*P<0.05).

MBC content of 0-10cm and 10-20cm soil with S800 treatment is higher than those of CK and S1600, No significant difference between S400, S800 and S1200. (Fig 2). The MBC contents were 168.75–204.17 mg kg^-1^ in the S_400_treatment, 160.00–229.50 mg kg^-1^ in the S_800_ treatment, 181.25–191.67 mg kg^-1^ in the S_1200_ treatment, and 135.42–157.08 mg kg^-1^ in the S_1600_ treatment. In the 10-20cm soil layer, the S_800_ treatment had the highest MBC content, which was significantly higher than that in the CK. The soil MBC content decreased in the following order: S_800_ > S_1200_, S_400_ > CK, S_1600_ at this depth. MBC in 20–40 cm is S1200, S400 greater than that of S1600 and CK.

The straw return to deep soil treatments significantly increased the MBC distribution at 0–20 cm compared with the CK. Similar phenomena were observed in the treatments with low and medium contents of MBC at 0–20 cm and 20–40 cm; however, this difference was not significant. The MBC contents at 0–10 cm and 10–20 cm were nearly identical (Fig 2).

### Effects of returning different amounts of straw to deep soil on easily oxidized organic carbon (EOC)

**Fig 3:**
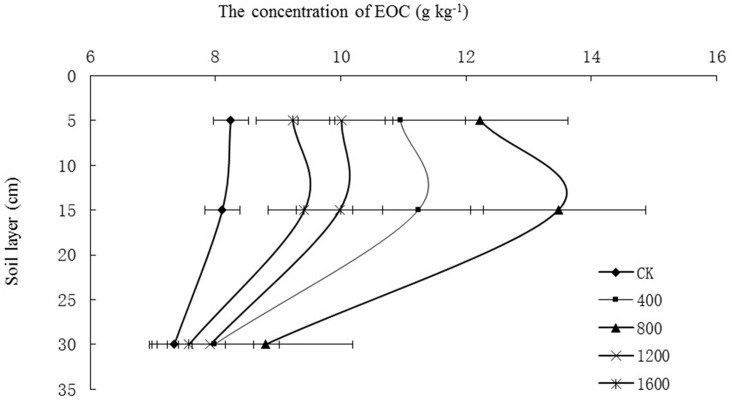
Effects of straw return to deep soil on the easily oxidized organic carbon (EOC) contents at three soil depths.

The straw return treatments increased the EOC at the three soil depths (Fig 3). The soil EOC content ranged from 7.98–11.23 g kg^-1^ for S_400_, 8.80–13.47 g kg^-1^ for S_800_, 7.92–10.02 g kg^-1^ for S_1200_ and 7.58–9.42 g kg^-1^ for S_1600_. The EOC contents in the S_800_ treatment were the highest, and the EOC contents in the CK were the lowest at each soil depth. Some appreciable differences were observed between the straw return in the different treatments, which decreased in the following order: S_800_ > S_400_ > S_1200_ > S_1600_ > CK (Fig 3).

The deep soil (depth of 20–40 cm) had the lowest EOC content compared with the other two depths. In the control treatment, the soil EOC decreased as the soil depth increased from 0–10 cm to 10–20 cm. In the straw return treatments, the soil EOC was higher at 10–20 cm than at 0–10 cm.

### Effects of returning different amounts of straw to deep soil on DOC

**Fig 4:**
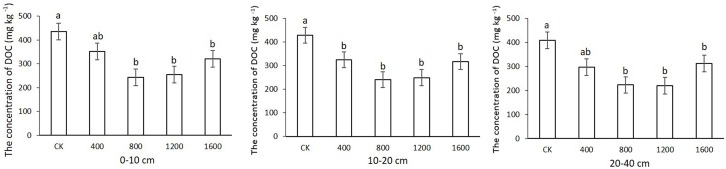
Effects of returning straw to the deep soil on soil dissolved organic carbon (DOC) at the three soil depths. Values are expressed as means±SE from three replicates. Different letters indicate significant differences (*P<0.05).

The soil DOC contents at the three investigated soil depths for different treatments are shown in Fig 4. The soil DOC ranged from 297.83–351.97 g kg^-1^ for S_400_, 223.08–243.88 g kg^-1^ for S_800_, 220–254.34 g kg^-1^ for S_1200_ and 311.83–321.43 g kg^-1^ for S_1600_. Straw return to deep soil significantly decreased the soil DOC. The straw return treatments had significantly lower soil DOC than the CK at each soil depth, except for the S_400_ treatment at depths of 0–10 cm and 20–40 cm. The soil DOC contents were not significantly different between the soil layers in each treatment. Regarding the vertical distribution of soil DOC, the soil DOC decreased with increasing soil depth in all treatments (Fig 4).

### The effects of different amounts of straw return on the soil light fraction organic carbon (LFOC)

**Fig 5:**
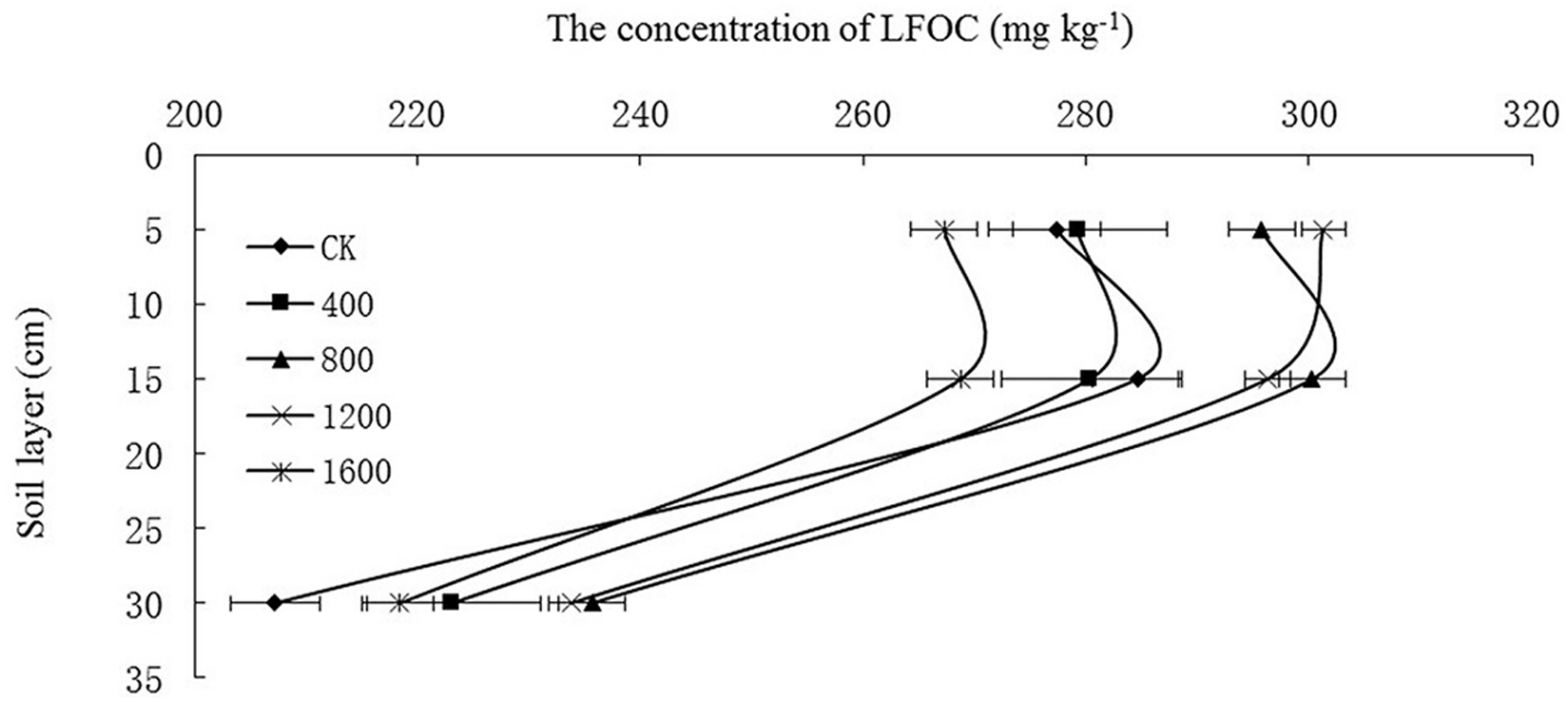
Effects of returning straw to deep soil on the soil light fraction organic carbon (LFOC) at three soil depths.

The soil LFOC contents at all soil depths are shown in Fig 5. The soil LFOC in the different straw return treatments ranged from 223.12–280.37 mg kg^-1^ for S_400_, 235.67–300.32 mg kg^-1^ for S_800_, 233.78–301.32 mg kg^-1^ for S_1200_, and 218.44–268.77 mg kg^-1^ for S_1600_. The ranges of soil LFOC in all treatments decreased as follows: S_1200_ > S_800_ > S_400_, CK >S_1600_ at 0–10 cm, S_800_, S_1200_ > CK, S_400_ > S_1600_ at 10–20 cm, and S_800_, S_1200_ > S_400_ > S_1600_ > CK at 20–40 cm (Fig 5).

## Discussion

After two years of straw return, the straw applied at 400 kg ha^-1^ (S400) and 800 kg ha^-1^ (S_800_) in the two districts was completely decomposed, and the straw in the other two treatments was partially decomposed. The quantity of straw return should be determined from the treatment with complete decomposition. In addition, many physical and chemical soil properties should be considered, especially the SOC content.

The C/N ratio is a key factor that influences the decomposition of crop straw[32]. High doses of crop straw should be applied to guarantee the absorption of available N by crops[26]. A C/N ratio of 25:1 facilitates the straw decomposition and the release of N[33]. Qiu *et al*. [25] and Ge *et al*. [34] found that using straw return and adjusting the C/N ratio could increase crop production. Therefore, in this paper, appropriate amounts of nitrogen fertilizer (C/N ratio of 25:1) were added after straw was returned to the fields.

Soil MBC is an active part of the SOC pool[29] and is influenced by easily degradable organic matter via processes such as the decomposition of microbial organisms, variations in soil moisture and soil management measures[31]. MBC participates in the decomposition and formation of soil organic matter, plays an important role in nutrient cycling and transformations, and serves as a repository for effective nutrients in the soil[35–37]. Straw return increased the MBC contents in the upper and deep soil layers. This increase in MBC could be explained by the increased availability of C and N. However, the MBC decreased in the S_1200_ and S_1600_ treatments relative to the S_800_ treatment. This decrease in microbial activity could be attributed to the soil pH changes caused by the large amounts of added urea[38]. Indeed, decreases in the soil pH following urea addition are suggested to be a main factor controlling decreases in MBC[39,40]. The results also indicated that the amount of urea added and the amount of straw return could be an important combined factor that affects the soil MBC content. These results were consistent with the results presented by Zhang *et al*. [41], who found that the microbial biomass and functional diversity decreased due to decreasing soil pH beyond a critical N loading level in a semi-arid temperate steppe. The threshold of the N loading level was 16 gNm^−2^year^−1^[42], which corresponded to the N-addition rates in the S_1200_ and S_1600_ treatments in this study.

Soil EOC may reflect the effectiveness of organic C for indicating soil quality, which was more sensitive than other indicators in farmland soil[30]. The EOC content was higher at 0–20 cm than at 20–40 cm because of increased microbial activity, and the soil permeability was improved after straw was returned to the 0-40-cm soil layer. The EOC content was the highest in S_800_, which could suggest that the application of 800 kg ha^-1^ straw was the most effective for improving soil microbial activity and the soil EOC content. The DOC content influenced the migration of soil organic and inorganic components and their transformations and degradation. In addition, the soil DOC content had a relatively strong impact on the migration of heavy metals and the adsorption of inorganic ions[42]. The soil DOC content was lower in the straw return treatments than in the CK. This result potentially occurred because the crops absorbed DOC during their growth and development or because the DOC was temporarily accumulated in or transformed to other substances. Otherwise, the decrease in DOC could be associated with microbial use[42]. In addition, the environmental changes in soil chemistry should also be considered[43–45]. The LFOC content was mainly dependent on the rates of organic matter input and decomposition and decreased as follows: S_800_> S_1200_> CK> S_400_> S_1600_. This result suggests that LFOC is an active form of organic carbon that reflects the variations in soil total organic C. Additionally, S_800_should facilitate the accumulation of LFOC.

In conclusion, returning 800kg ha^-1^of straw to the soil with urea application generally enhanced the EOC, MBC, and LFOC contents and was a suitable agricultural practice in this region for the new straw return method.
